# Amyloid-beta relates to default and attention network connectivity during working memory retrieval

**DOI:** 10.1093/braincomms/fcag278

**Published:** 2026-07-16

**Authors:** Hengda He, Christian G Habeck, Yaakov Stern

**Affiliations:** Cognitive Neuroscience Division, Department of Neurology, Vagelos College of Physicians and Surgeons, Columbia University, New York, NY 10032, USA; Cognitive Neuroscience Division, Department of Neurology, Vagelos College of Physicians and Surgeons, Columbia University, New York, NY 10032, USA; Taub Institute for Research on Alzheimer’s Disease and the Aging Brain, Columbia University, New York, NY 10032, USA; Gertrude H. Sergievsky Center, Columbia University, New York, NY 10032, USA; Cognitive Neuroscience Division, Department of Neurology, Vagelos College of Physicians and Surgeons, Columbia University, New York, NY 10032, USA; Taub Institute for Research on Alzheimer’s Disease and the Aging Brain, Columbia University, New York, NY 10032, USA; Gertrude H. Sergievsky Center, Columbia University, New York, NY 10032, USA

**Keywords:** amyloid, task connectivity, fMRI, working memory

## Abstract

As one of the earliest pathological events in Alzheimer’s disease, amyloid-beta accumulation has been linked to alterations in large-scale brain networks, especially the default mode network. While most prior work has focused on resting-state functional connectivity or task-evoked activation, how amyloid-beta relates to task-modulated connectivity that directly supports behaviour is less explored. Here, we examined the relationship between amyloid-beta burden and task-modulated connectivity during a Letter–Sternberg verbal working memory task in 84 cognitively normal older adults from an ongoing longitudinal cohort (aged 56–71 years; 41 females). To further understand these connectivity alterations, we tested whether these connections were associated with task performance or with cognitive reserve proxies. We found that higher amyloid-beta burden was associated with reduced connectivity—specifically during the probe-phase of working memory retrieval. At the regional scale, lower node strength in posterior–medial regions of the default mode network was related to worse task performance and mediated the amyloid–behaviour relationship. At the network scale, altered coupling between the default mode and dorsal attention networks was related to task performance but did not mediate the amyloid–behaviour relationship. Moreover, physical activity was associated with network-level coupling but not with regional node strength. These findings suggest that amyloid-beta burden disrupts retrieval-related working memory systems, particularly regions of the default mode network, while coupling between the default mode and dorsal attention networks is related to physical activity, a cognitive reserve proxy. Collectively, task-modulated connectivity provides insight into pathways through which early Alzheimer’s disease pathology influences working memory performance and may inform targeted lifestyle or neuromodulation interventions for older adults.

## Teaser

He *et al.* report that greater amyloid-beta burden was linked to reduced working memory connectivity during retrieval, with lower posterior–medial default mode network node strength mediating worse performance. Amyloid-related default mode and dorsal attention network coupling was associated with task performance and physical activity (a cognitive reserve proxy).

## Graphical Abstract

**Graphical Abstract:**
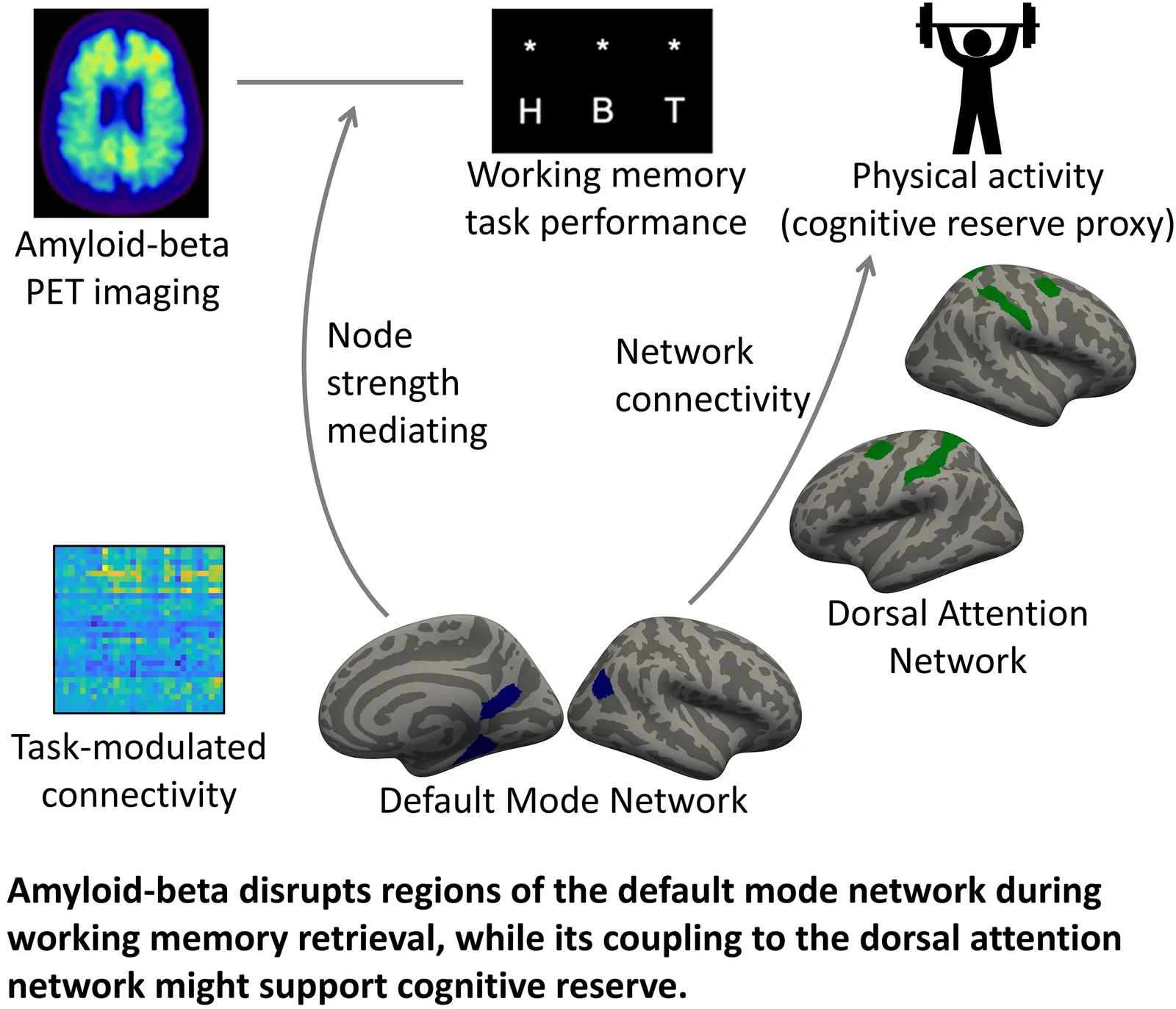

## Introduction

Many older adults remain cognitively normal despite accumulating pathological proteins in the brain. However, the build-up of amyloid-beta (Aβ) and tau aggregates^1^ is associated with an increased risk of future cognitive decline,^2^ marking the preclinical phase of Alzheimer’s disease.^3^ Among these pathological processes, Aβ deposition is considered one of the earliest initiating events in the disease cascade and can trigger downstream neuropathological changes, including tau propagation, synaptic and network dysfunction and ultimately cognitive impairment.^4,5^ Growing evidence indicates that large-scale functional brain networks, particularly the default mode network (DMN), are vulnerable to such early pathological disruptions, with such alterations potentially linked to cognitive decline.^6-8^ However, it is not fully understood how early Alzheimer’s disease pathology, such as Aβ, interacts with functional brain networks during task performance. Furthermore, individual differences in cognitive reserve proxies, including education, IQ and physical activity (PA), may also influence brain network alterations or adaptations in the presence of Aβ pathology,^9-12^ but the functional pathways underlying these mechanisms are not fully established. Cognitive reserve refers to a property of the brain that allows for cognitive performance that is better than expected given the degree of life-course-related brain changes and brain injury or disease.^13^ Because cognitive reserve cannot be measured behaviourally, human studies typically rely on proxy measures, such as education, IQ and lifestyle factors. Here, education, IQ and PA were treated as cognitive reserve proxies. Education and IQ are commonly used cognitive reserve proxies.^14-17^ More recently, PA has been identified as a modifiable lifestyle factor that contributes to cognitive reserve.^18^ We examined how Aβ burden relates to task-modulated functional connectivity during a working memory (WM) task and tested how these Aβ-associated connectivity changes relate to cognitive performance and cognitive reserve proxies in cognitively normal older adults.

Recent studies have demonstrated that early Aβ deposition preferentially accumulates in and affects the DMN, especially its highly connected hub regions, representing regional vulnerability to early Alzheimer’s disease pathology.^19-21^ However, most existing neuroimaging findings are based on resting-state functional MRI (fMRI) and correlation-based connectivity measures, which limit interpretation regarding whether altered connectivity reflects pathological vulnerability or adaptive compensation.^22^ Task-modulated connectivity derived from task-based fMRI captures dynamic, context-dependent interactions among brain regions, which are distinct from correlation-based estimates of spontaneous fluctuations.^23^ Recent evidence using task-based connectivity approaches suggests that Aβ-related alterations in network connectivity may influence downstream neuropathological processes, including tau accumulation.^24,25^ In addition to Aβ-related hub region vulnerability, the functional activity of the DMN and dorsal attention network (DAN) is implicated in cognitive reserve mechanisms, where these networks may adaptively compensate for the impact of pathology or neurodegeneration on cognition.^26,27^ Therefore, alterations in these large-scale networks during ageing and early Alzheimer’s disease may represent both local vulnerability to pathology and potential mechanisms that support cognitive reserve.^13^ Accordingly, brain–behaviour relationships related to Aβ burden may be more tightly linked to regional hub connectivity, whereas associations with cognitive reserve proxies may emerge more prominently at the network level. We therefore hypothesized that Aβ-related disruptions in DMN hub connectivity are associated with poorer task performance, while DMN couplings with the DAN could be related to cognitive reserve processes.

WM refers to the cognitive systems responsible for the temporary maintenance and manipulation of information, that are critical for supporting higher-order functions such as reasoning, decision-making and goal-directed behaviour.^28^ WM is known to decline with ageing and Alzheimer’s disease^29^ and is disrupted in the early stages of Alzheimer’s disease.^30^ In the current study, participants performed a Letter–Sternberg (LTS) verbal WM task during fMRI, which included three task phases: encoding, retention and probe/retrieval, as well as three memory load conditions. Previous studies have shown that Aβ pathology influences WM-related functional activity. For example, Oh *et al*.^31^ reported Aβ-associated load-dependent hyperactivity in the frontoparietal network (FPN) during a WM task, and Kennedy *et al*. found that Aβ burden interacts with WM-related brain function in a nonlinear fashion, affecting both task performance and executive control.^32^ More recently, Argiris *et al*.^33^ demonstrated that phase-specific patterns of WM functional activation across encoding, retention and probe may represent a brain maintenance mechanism in the presence of neurodegeneration. These findings highlight the importance of examining WM task phases separately. However, it remains unclear which specific phase of WM is most affected by Aβ pathology and how such effects contribute to cognitive performance. Furthermore, examining Aβ-related alterations in connectivity and their relationships to task performance, as well as to cognitive reserve proxies, has important implications for early intervention design, such as identifying optimal network targets for transcranial magnetic stimulation (TMS) to enhance cognitive functioning^34,35^ and establishing connectivity-based markers to monitor the progression and efficacy of interventions.^36,37^

In this study, we first examined task-modulated functional connectivity during a WM task using a whole-brain psychophysiological interaction (PPI) analysis.^38^ We then investigated how Aβ burden, as estimated from PET imaging, was related to task-modulated connectivity across different phases of the task. To assess whether Aβ-related connectivity alterations were associated with task performance or with cognitive reserve proxies, we quantified WM efficiency, calculated as accuracy divided by response time (RT), and assessed the cognitive reserve proxies IQ, education and PA. To test whether Aβ-related effects differ across spatial scales, we examined task-modulated connectivity at both the regional and large-scale network levels. We hypothesized that higher Aβ burden would be related to reduced connectivity of DMN hub regions, which in turn would be associated with poorer task performance. Furthermore, we hypothesized that higher network-level connectivity, particularly between the DMN and DAN, would be related to higher cognitive reserve proxy measures, reflecting adaptive network reconfiguration in the presence of Aβ pathology. Together, this study provides insight into how Aβ burden disrupts WM-related connectivity and identifies potential neural processes that support cognitive reserve.

## Materials and methods

### Participants and study design

Participants were drawn from the ongoing Cognitive Reserve/Reference Ability Neural Network (CogRes/RANN) longitudinal study.^39^ Recruitment was conducted through random market mailings in the New York metropolitan area. Individuals were screened to ensure they were right-handed, native English speakers with no major neurological or psychiatric disorders, history of head injury, significant medical illness or hearing/vision impairment that could interfere with MRI acquisition or cognitive testing. All participants were cognitively normal at data acquisition, and dementia or mild cognitive impairment (MCI) was excluded using the Dementia Rating Scale,^40^ with a minimum inclusion score of 130. The experimental design of our study and the recruitment process were approved by the Internal Review Board of the College of Physicians and Surgeons of Columbia University. All participants have provided informed consent to participate in the study, and written consent was obtained from them. For the present analysis, we included subjects who had completed baseline structural and task-based functional MRI and amyloid PET imaging and had available demographic data.

Leisure-time PA was assessed at the baseline visit using a modified version of the Godin Leisure-Time Exercise Questionnaire,^41^ which asked participants to recall their PA over the past week. Specifically, it was assessed using a self-reported exercise questionnaire that recorded the weekly frequency of strenuous, moderate and mild exercise. Standard metabolic equivalent of task (MET) values were assigned to each intensity category (nine for strenuous, five for moderate and three for mild activity). Total weekly METs were calculated as the sum of three category-specific scores derived from exercise frequency, estimated duration and the corresponding MET value. Specifically, strenuous, moderate and mild exercises were weighted by factors of 0.5, 0.75 and 1 h, respectively, before multiplication by their MET values. The total MET measure was used as the PA variable in the analyses. IQ was assessed using the American National Adult Reading Test (AMNART).

### Letter–Sternberg working memory task paradigm and behavioural measure

**Figure 1:**
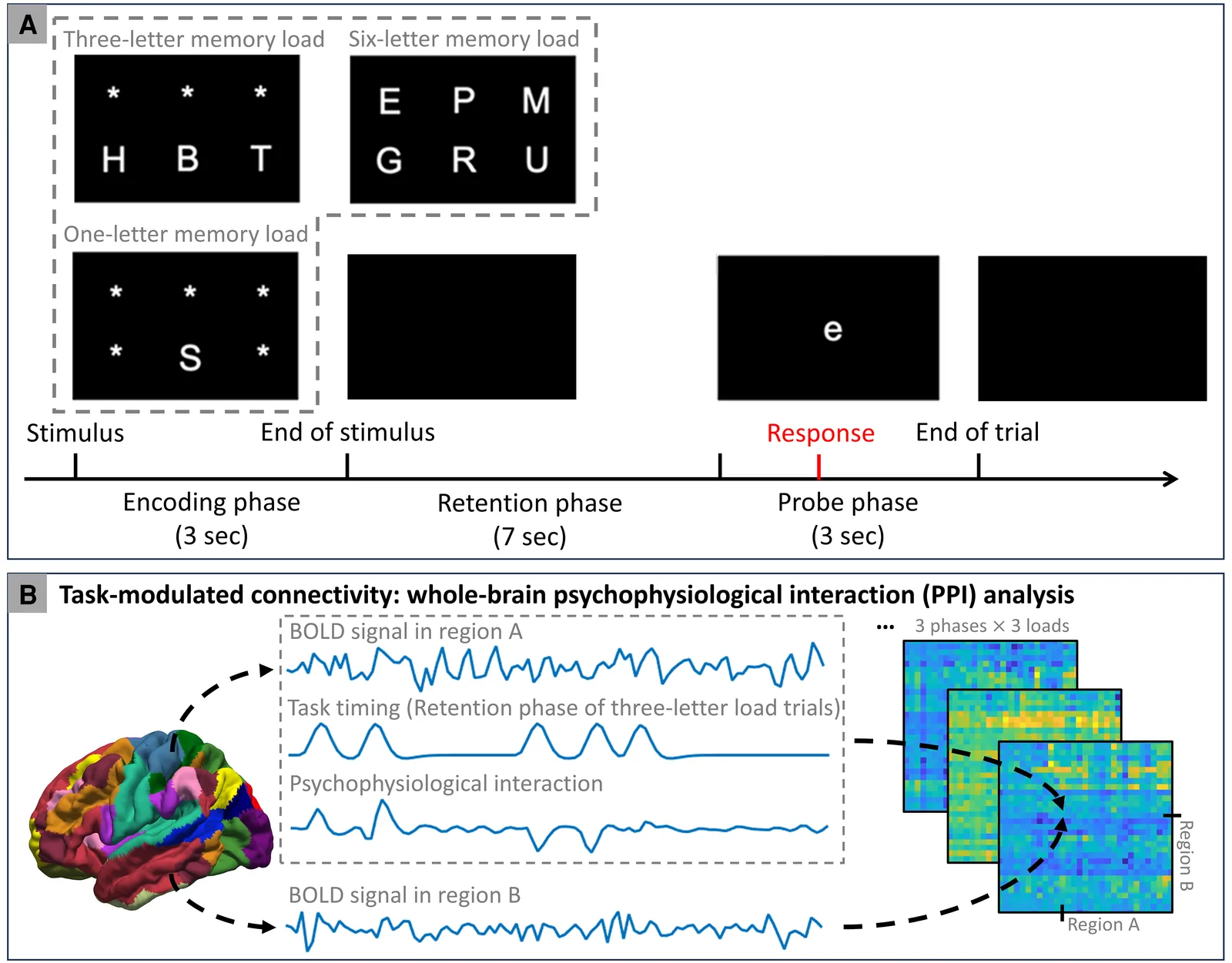
**Functional MRI task paradigm and task-modulated functional connectivity analysis**. (**A**) LTS verbal working memory task paradigm. Each trial started with a presentation of an upper-case letter set for 3 s (encoding phase), followed by a 7 s blank screen (retention phase), and a 3 s presentation of a lower-case probe letter (retrieval phase). Participants were instructed to press the button indicating whether the probe letter was in the letter set during the encoding phase. Memory load (one, three or six letters for encoding) was randomized across trials. Task performance was quantified as efficiency (accuracy divided by median RT). (**B**) Task-modulated PPI functional connectivity analysis. Whole-brain PPI models were used to estimate connectivity between regions of interest from the Schaefer atlas. For each participant, nine connectivity matrices (3 phases × 3 loads) were estimated by modelling the interaction between regional BOLD signals and WM task timing, controlling for task-evoked activation and nuisance covariates.

As illustrated in Fig. 1A, during fMRI acquisition, participants performed an LTS verbal WM task with three memory load conditions (one, three or six letters). Each trial began with the presentation of an upper-case letter set for 3 s (encoding phase), followed by a 7 s of blank screen (retention phase), and ended with a lower-case probe letter displayed for 3 s (probe/retrieval phase). Participants indicated by button press whether the probe letter was included in the original set. Each participant completed 90 trials across three fMRI runs, with a jittered inter-trial interval (gamma distribution; *k* = 1.3, *θ* = 2) to randomize trial onset. Participants were instructed to respond as accurately and quickly as possible and were trained on the task before scanning. Responses were collected with a fibre-optic button box by button press. The primary behavioural measure of LTS WM tasks was WM efficiency. This task performance measure was calculated as accuracy divided by median RT and then averaged across three memory loads. Mean accuracy and median RT were also examined separately.

### MRI and PET data acquisition

All MRI data were collected on a 3 T Philips Achieva Magnet scanner. Anatomical high-resolution T1-weighted images were acquired using a magnetization-prepared rapid gradient echo sequence [TR/TE = 6.6/3.0 ms; flip angle = 8°; field of view (FOV) = 256 × 256 mm; matrix = 256 × 256; voxel size = 1 mm isotropic; 165 axial slices]. A neuroradiologist confirmed the absence of clinically significant abnormalities in all participants. LTS WM task-based fMRI scans were collected with a T2*-weighted echo-planar imaging (EPI) sequence (TR/TE = 2000/20 ms; flip angle = 72°; FOV = 224 × 224 mm; matrix = 112 × 112; slice thickness = 3 mm; 36 axial slices, 314 volumes). Three runs of ∼10.5 min each were collected. The first three volumes of each run were discarded to allow for magnetization equilibrium.

Amyloid PET imaging used [18F]Florbetaben (donated by Piramal Pharma, Inc.) tracer on a Siemens Biograph 64 mCT/PET scanner (3D dynamic acquisition, four 5-min frames, 20 min total). Data acquisition began 50 min after a 10 mCi dose bolus injection of 18F-florbetaben. A structural CT scan (0.58 × 0.58 mm in-plane resolution; 3 mm slice thickness) was also acquired for attenuation correction purposes.

### MRI and PET data processing

Functional MRI preprocessing was performed using FSL (v6.0.4) and custom Python code. The pipeline included motion correction (MCFLIRT^42^) with the first volume as the reference volume, slice-timing correction with *slicetimer*,^43^ temporal high-pass filtering (>0.01 Hz) to remove scanner drift and spatial smoothing with a 5 mm full-width at half-maximum Gaussian kernel. Structural T1-weighted images were processed with FreeSurfer (v5.1) for tissue segmentation and cortical surface reconstruction. Cortical parcellation was performed with the Schaefer atlas^44^ (200 cortical parcels with 17 networks version). Specifically, the surface-space Schaefer parcellation was registered to everyone’s cortical surface space and projected to the native EPI volumetric space. Tissue segmentation and registrations were visually inspected for quality control.

Amyloid PET images were processed with an in-house-developed pipeline.^45^ Briefly, PET frame images were registered to the first frame image with rigid-body transformation and averaged to generate a static PET image. Then this image was co-registered to each participant’s CT image to create a PET-CT composite image. This image was later registered to each participant’s structural T1-weighted brain image. The Schaefer parcellation regions of interest (ROIs) and the cerebellar grey-matter mask (FreeSurfer segmentation) were transformed into the PET image space. Lastly, standardized uptake value ratios (SUVRs) were calculated for each ROI by normalizing regional tracer uptake to the cerebellar grey-matter reference. Aβ burden was calculated as the mean SUVR across cortical regions previously identified as being linked to longitudinal cognitive decline in global cognition (defined as Aβ signature regions; more details are provided in a previous study^46^; regions include temporo-occipital, orbitofrontal and ventrolateral prefrontal cortex). A global SUVR was also computed across the entire cortex.

### Task-modulated functional connectivity

To examine the task-modulated functional connectivity during the WM process, we performed a whole-brain PPI analysis (Fig. 1B). For each participant, we first extracted the blood oxygenation level-dependent (BOLD) time series of every cortical region defined by the Schaefer parcellation. Task-modulated connectivity was then estimated using multiple regression, where the BOLD signal of each region (ROI A) was modelled as a linear combination of (i) the BOLD signal of a second region (ROI B); (ii) task-timing regressors representing the onsets and durations of the three WM task phases (encoding, retention, and probe) at each of the three memory loads (one-, three- and six-letter sets); (iii) the PPI interaction terms formed by multiplying the deconvolved BOLD signal of ROI B with each of the nine phase-by-load task boxcar functions, and re-convolving with the canonical haemodynamic response function; and (iv) controlling for nuisance covariates (motion parameters, large motion volumes, mean BOLD signals from white matter and lateral ventricles and constant terms for each run). The inclusion of task-timing regressors ensured that task-evoked mean activation was controlled, thereby isolating task-modulated connectivity effects from task co-activation. To further control potential false-positive results,^47^ FMRIB’s linear optimal basis set (FLOBS)^48^ was used to model task-timing regressors. Specifically, a boxcar function representing the timing of task trials for each phase and load was convolved with the FLOBS basis set, yielding a total of 27 task-timing regressors. For each subject, the PPI model resulted in nine task-modulated connectivity matrices (three phases by three loads). Beta coefficients of the interaction terms represented the strength of task-modulated connectivity between each pair of regions. Lastly, we also calculated positive and negative node strength, as the sum of all positive or negative edge weights associated with each ROI.

### Statistical analysis

All analyses controlled for age and sex. We first tested the relationship between Aβ burden and LTS WM task performance. Average Aβ SUVR in the signature regions was correlated with WM task efficiency performance, RT and accuracy, using partial Spearman correlation and controlling for covariates. Because education was treated as a cognitive reserve proxy and a variable of interest in this study, it was not included as a confounder in the primary analyses.

To investigate whether task-modulated connectivity exhibits phase-dependent or load-dependent effects, repeated-measures ANOVA tests were used on edgewise PPI connectivity, with false discovery rate (FDR) correction at *q* < 0.05 across edges. Group-level task-modulated connectivity for each task phase was computed using a one-sample *t*-test (FDR-corrected *P* < 0.05) with connectivity matrices averaged across the loads.

Next, the task-modulated connectivity was related to amyloid burden. For each phase, partial Spearman correlations were used to assess associations between amyloid SUVR and PPI connectivity averaged across memory loads. Significant effects were identified with FDR correction (*q* < 0.05) across edges between pairs of ROIs. Then, positive and negative node strength was also related to amyloid burden, and significant relationships were identified with FDR correction. Lastly, node-level connectivity was summarized into network-level connectivity based on the network label of each node from the Schaefer atlas network assignment. Network-level connectivity of each phase was correlated with the amyloid burden, and significant edges were extracted with FDR-corrected *P* < 0.05.

To assess the associations between amyloid-related connectivity and behavioural outcomes, we implemented a 20-fold cross-validation (CV) framework with 500 random repetitions. This repeated *K*-fold strategy was chosen because it provides more stable and less biased estimates than leave-one-out approaches, particularly in moderate sample sizes.^49^ Specifically, in each repetition, participants were randomly partitioned into 20 folds. For each fold, a linear regression model was trained on 19 folds using the amyloid-related node- or network-level averaged connectivity measures as a predictor of task performance, with age and sex included as covariates. The trained model was then applied to the held-out fold to generate out-of-sample predictions. This process was iterated until every fold was used as the test set once, resulting in predicted values for all participants. The entire 20-fold CV procedure was repeated 500 times with different random partitions to minimize variability due to data splits. Predictive performance was quantified as the median Pearson correlation coefficient between predicted and observed outcomes across repetitions. To assess the significance of the cross-validated correlation, permutation testing (1000 iterations) was used by randomly shuffling the dependent variable to generate a null distribution. The *P*-value was computed as the proportion of permutations that were greater than or equal to the observed cross-validated correlation. CV frameworks with different selection of fold number *K* were also tested, and results were reported in the Supplementary Text. The same repeated 20-fold cross-validation procedure was used to examine whether amyloid-related node- and network-level connectivity was associated with cognitive reserve proxies, including IQ, education and PA, with age and sex included as covariates.

For the subgroup of participants with follow-up data available (*N* = 22; mean age = 65.09 years, SD = 3.60; 11 female), we performed a longitudinal analysis to examine the association between baseline PA and subsequent change in amyloid-related connectivity. Specifically, partial Pearson correlation analyses were conducted, controlling for age, sex and baseline amyloid-related connectivity.

For task-modulated connectivity measures that demonstrated a significant relationship to task performance, we tested whether these connectivity measures mediated the influence of amyloid burden on WM task performance. A bootstrapped mediation model was used with 5000 resamples, and we reported 95% confidence intervals (CIs) and two-tailed *P*-values for both the indirect mediated and direct effects. Moderation analyses were performed to test the interaction effect of amyloid burden and connectivity on task performance. The model included the main effects of amyloid burden and connectivity measures, their interaction term and covariates. Moderation was assessed by evaluating the significance of the amyloid-by-connectivity interaction term on task performance.

## Results

### Study participants

Eighty-four participants from the CogRes/RANN study with available datasets were included in the analyses (age range 56–71 years; mean ± standard deviation (SD) = 65.5 ± 3.4; 41 females), with education ranging from 12–20 years (mean ± SD = 16.3 ± 2.2). All participants performed the LTS WM task well, with a mean WM efficiency of 0.87 (SD = 0.20, range from 0.54 to 1.53), mean RT of 1.19 s (SD = 0.24, range from 0.66 to 1.68) and mean accuracy of 0.95 (SD = 0.04, range from 0.78 to 1.00). The mean global amyloid SUVR among the participants was 1.16 (SD = 0.09) with 3 out of 84 participants classified as amyloid positive using a cut-off of 1.34 as defined from a previous study.^50^ The mean amyloid SUVR within the signature regions was 1.09 (SD = 0.08), including 1.13 (SD = 0.10) in the left temporal–occipital region, 1.07 (SD = 0.10) in the left orbital frontal cortex and 1.06 (SD = 0.10) in the left lateral ventral prefrontal cortex. IQ data were available for 82 participants, and PA data were available for 68 participants.

### Association of amyloid burden and working memory task performance

We first examined whether amyloid burden in signature regions was related to WM task performance. The primary behavioural outcome was task efficiency, calculated as accuracy divided by median RT and averaged across memory loads. Higher signature region amyloid SUVR was significantly associated with poorer task performance (*r* = −0.3237, *P* < 0.0032, controlling for age and sex). We also tested global SUVR and individual performance components. A similar negative association was observed for global amyloid SUVR (*r* = −0.2348, *P* < 0.0349). Higher amyloid burden was further related to slower RTs (signature: *r* = 0.3219, *P* < 0.0034; global: *r* = 0.2310, *P* < 0.0380) and lower accuracy (signature: *r* = −0.2773, *P* < 0.0122; global: *r* = −0.1996, *P* = 0.0740).

### Task-modulated connectivity supporting working memory

**Figure 2:**
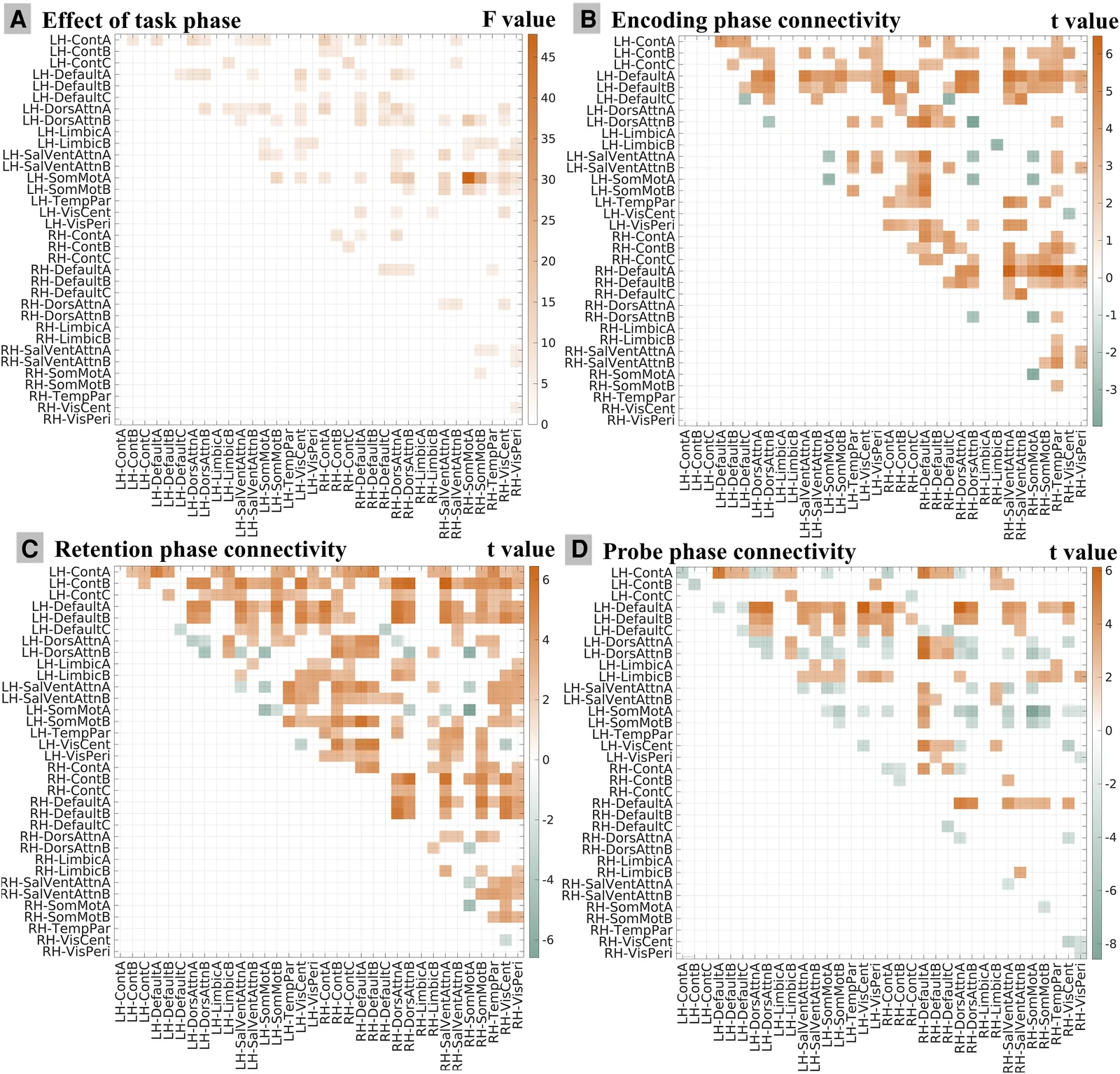
**Task-modulated PPI connectivity during the LTS WM task**. (**A**) Repeated-measures ANOVA showing the main effect of task phase (encoding, retention, probe) on PPI connectivity across all memory loads (*F* values, FDR-corrected *P* < 0.05). (**B–D**) Significant group-level task-modulated connectivity (*t* values) identified for the encoding (**B**), retention (**C**) and probe (**D**) phases. Positive *t* values indicate increased connectivity during the task relative to baseline, whereas negative *t* values indicate decreased connectivity. Connectivity matrices represent pairwise connections across left hemisphere (LH) and right hemisphere (RH) cortical networks based on the Schaefer atlas brain parcellation. Data from 84 participants were included in the statistical test. Cont, cognitive control network; Default, default mode network; DorsAttn, dorsal attention network; Limbic, limbic network; SalVentAttn, salience/ventral attention network; SomMot, somatomotor network; TempPar, temporoparietal network; VisCent, visual central network; VisPeri, visual peripheral network.

We next examined task-modulated connectivity using PPI analysis. For each subject, nine connectivity matrices were derived, reflecting the three memory loads (one-, three- and six-letter sets) across the three task phases (encoding, retention, and probe). A repeated-measures ANOVA revealed a significant main effect of phase on task-modulated connectivity (FDR-corrected *P* < 0.05; Fig. 2A), but no significant effect of memory load. Accordingly, connectivity measures were averaged across memory load conditions for subsequent analyses. We then identified significant group-level connectivity for each phase: (i) during encoding, the strongest positive edge was observed between RH-SalVentAttnA (right hemisphere salience/ventral attention subnetwork A) and RH-DefaultA (default mode subnetwork A) networks (*t* = 6.4832, *P* < 6.0753e−09, FDR-corrected *P* < 2.1051e−06), and the strongest negative edge between RH-DorsAttnB (dorsal attention subnetwork B) and LH-DorsAttnB (left hemisphere dorsal attention subnetwork B) (*t* = −3.9790, *P* < 1.4743e−04, FDR-corrected *P* < 0.0016) (Fig. 2B); (ii) during retention, the strongest positive edge was between RH-SalVentAttnA and LH-ContB (cognitive control subnetwork B) (*t* = 6.4515, *P* < 6.9861e−09, FDR-corrected *P* < 2.0784e−06), and the strongest negative edge between RH-SomMotA [somatomotor (SMN) subnetwork A] and LH-SomMotA networks (*t* = −6.5912, *P* < 3.7739e−09, FDR-corrected *P* < 2.0784e−06) (Fig. 2C); and (iii) during the probe phase, connectivity was most positive between LH-DefaultA and LH-VisCent (visual central network) (*t* = 6.1267, *P* < 2.8774e−08, FDR-corrected *P* < 5.7069e−06) and most negative between RH-SomMotA and LH-SomMotA networks (*t* = −8.5751, *P* < 4.6265e−13, FDR-corrected *P* < 2.7528e−10) (Fig. 2D).

### Amyloid burden is associated with altered probe-phase connectivity

**Figure 3:**
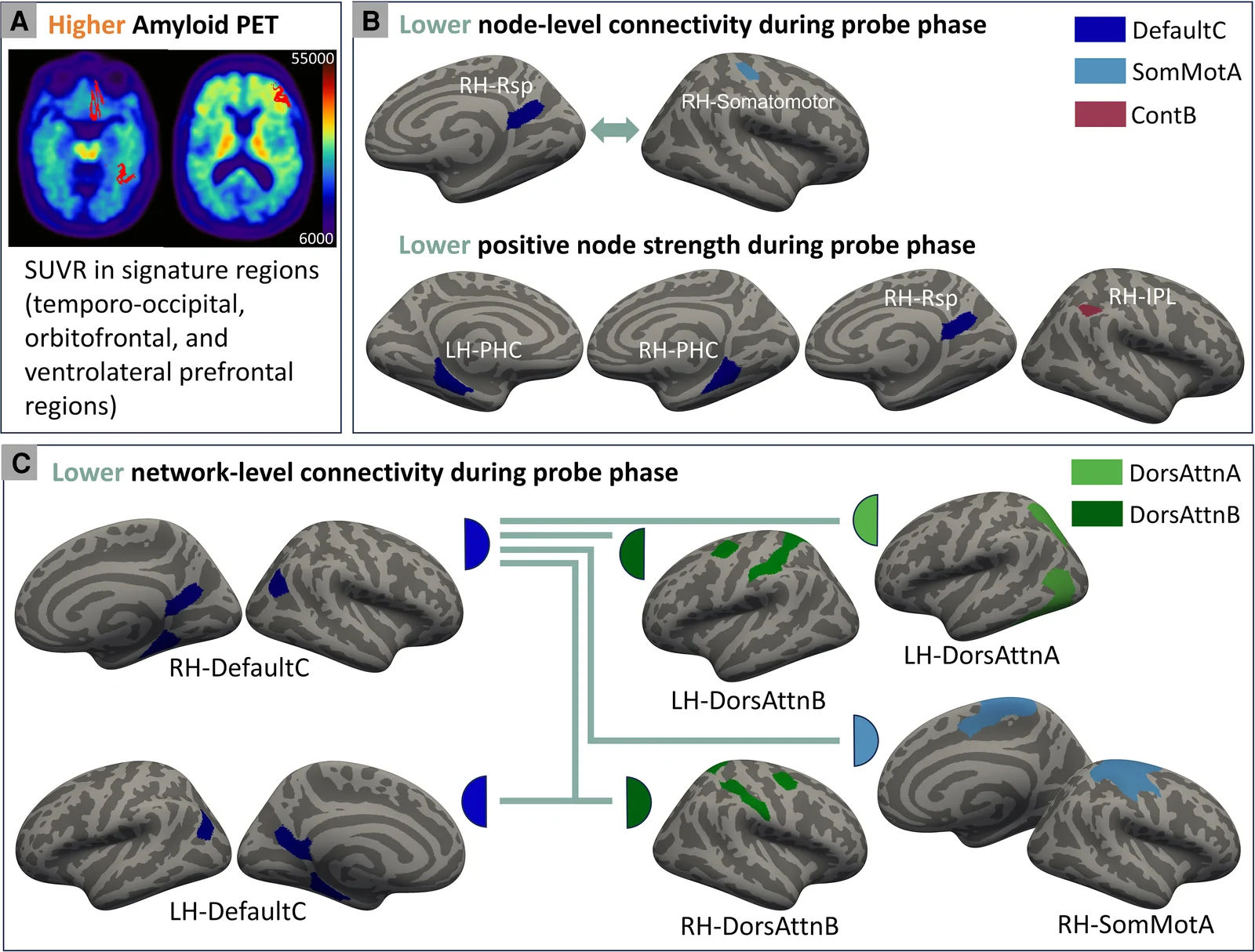
**Amyloid-associated changes in probe-phase task-modulated connectivity**. (**A**) PET images were used to capture amyloid burden within the predefined signature regions (highlighted in red; encompassing the temporo-occipital, orbitofrontal and ventrolateral prefrontal cortices). The heatmap scale represents raw image intensity in arbitrary units (a.u.). (**B**) Node-level amyloid-connectivity associations during the probe phase. Higher amyloid-beta burden was linked to lower connectivity between the RH-Rsp and the right somatomotor area (RH-Somatomotor) (*r* = −0.5053, *P* < 1.4980e−06, FDR-corrected *P* < 0.0298) and to lower positive node strength in the bilateral parahippocampal cortex (LH-PHC, RH-PHC) (LH-PHC: *r* = −0.4610, *P* < 1.4859e−05, FDR-corrected *P* < 0.0030; RH-PHC: *r* = −0.3999, *P* < 2.1697e−04, FDR-corrected *P* < 0.0217), RH-Rsp (*r* = −0.3791, *P* < 4.8275e−04, FDR-corrected *P* < 0.0277) and the RH-IPL (*r* = −0.3754, *P* < 5.5467e−04, FDR-corrected *P* < 0.0277). (**C**) Network-level amyloid-connectivity associations during the probe phase. Higher amyloid-beta was associated with lower connectivity between the default mode network (Default) and both the dorsal attention (DorsAttn) and the somatomotor (SomMot) networks (RH-DefaultC and LH-DorsAttnA: *r* = −0.3918, *P* < 2.9800e−04, FDR-corrected *P* < 0.0355; RH-DefaultC and LH-DorsAttnB: *r* = −0.4884, *P* < 3.7321e−06, FDR-corrected *P* < 0.0011; RH-DefaultC and RH-DorsAttnB: *r* = −0.4271, *P* < 6.9927e−05, FDR-corrected *P* < 0.0139; RH-DefaultC and RH-SomMotA: *r* = −0.4885, *P* < 3.7214e−06, FDR-corrected *P* < 0.0011; RH-DorsAttnB and LH-DefaultC: *r* = −0.4071, *P* < 1.6241e−04, FDR-corrected *P* < 0.0242). Spearman correlations were used with data from 84 participants.

We tested whether amyloid SUVR in signature regions was related to task-modulated connectivity across phases of the WM task, with connectivity averaged across memory loads. No significant associations were observed for encoding or retention phases. In the probe phase, however, higher amyloid burden was associated with reduced connectivity at both the region and network levels (Fig. 3). At the node level (Fig. 3B), higher amyloid was linked to reduced connectivity between the right retrosplenial area (RH-Rsp) and the right SMN area, as well as lower positive node strength in the bilateral parahippocampal cortex (PHC), the RH-Rsp and the right inferior parietal lobule (RH-IPL). At the network level (Fig. 3C), higher amyloid was associated with reduced connectivity between the DMN and both the DAN and SMN network (specifically, between the bilateral DMN and the bilateral DAN and between the right DMN and the right SMN network). Analyses using global amyloid SUVR showed convergent but weaker effects (Supplementary Tables 1 and 2). For comparison purposes, relationships between amyloid burden and resting-state functional connectivity were also tested, and no significant relationship was observed with FDR-corrected *P* < 0.05 (more details in Supplementary Text and Fig. 1).

### Regional node strength mediates the association between amyloid-beta burden and task performance

**Figure 4:**
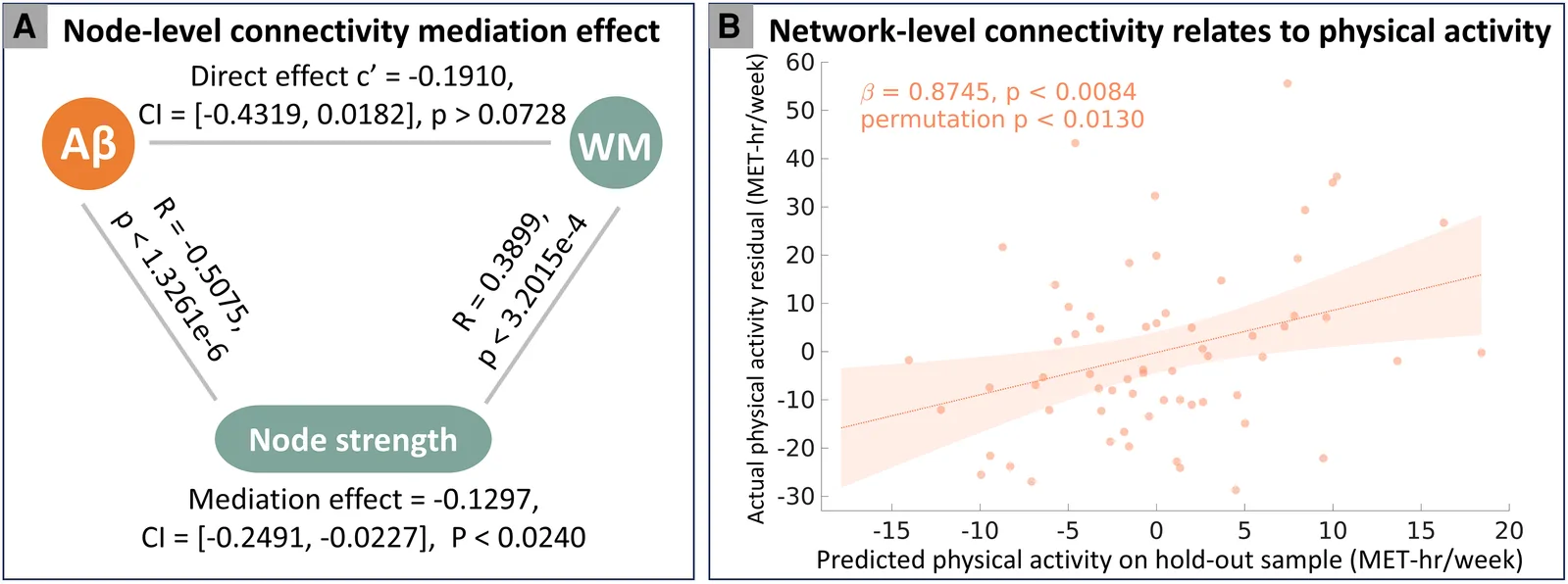
**The relationships between WM task performance, PA cognitive reserve proxy and Aβ-related node-level and network-level connectivity**. (**A**) Mediation analysis demonstrated a significant mediation effect of node-level connectivity during WM retrieval in the relationship between Aβ and WM task performance. Data from 83 participants were included in the statistical test. (**B**) Cross-validated prediction of PA from Aβ-related network-level connectivity. Connectivity between DMN and DAN significantly predicted PA. The shaded area indicates the 95% CI of the regression line. Data from 68 participants were included in the statistical test. Each data point represents one participant.

We next tested whether regional node strength was related to task performance. Positive node strength values from the regions identified in the previous analysis (Fig. 3B) were averaged and included in a mediation model. Higher averaged node strength was positively associated with better task performance (*r* = 0.3899, *P* < 3.2015e−04, Spearman correlation controlling age and sex). Mediation analysis indicated a significant indirect effect of amyloid burden on task performance through regional node strength [mediation effect = −0.1297, 95% CI = (−0.2491 to −0.0227), *P* < 0.0240], while the direct effect of amyloid on task performance was not significant [*c*′ = −0.1910, 95% CI = (−0.4319–0.0182), *P* = 0.0728] (Fig. 4A). Analyses using global amyloid SUVR showed a similar mediation effect (mediation effect *P* < 0.0064; Supplementary Text). No significant moderation effect of connectivity node strength on the relationship between amyloid burden and task performance was observed (testing on the interaction term: *β* = 0.0432, *t* = 1.5725, *P* = 0.1199). Amyloid-associated connectivity between the RH-Rsp and the right SMN area (Fig. 3B) was not significantly related to task performance (*r* = 0.2097, *P* = 0.0602).

We also tested the relationship between regional node strength and task performance using cross-validation. Averaged node strength values from the identified regions were used as an independent variable in a 20-fold cross-validation framework with 500 repeats and permutation testing. Regional node strength was significantly related to task performance (permutation *P* < 0.0020), with results consistent across different cross-validation folds (Supplementary Text). Similarly, the averaged network connectivity from the identified edges (Fig. 3C) was also significantly related to task performance (cross-validation permutation *P* < 0.0150). However, averaged network connectivity did not significantly mediate the effect of amyloid burden on task performance (mediation effect = −0.0656, 95% CI = (−0.1760–0.0408), *P* = 0.1772].

### Amyloid-related network-level connectivity is associated with physical activity

We examined whether amyloid-related network-level connectivity was associated with proxies of cognitive reserve, including education, IQ and PA. Cross-validation analyses showed no significant associations between averaged network-level connectivity and education (permutation *P* = 0.6903) or IQ (permutation *P* = 0.3836). Amyloid-associated network-level connectivity was significantly related to PA (permutation *P* < 0.0130; Fig. 4B). However, network-level connectivity did not significantly moderate the effect of amyloid burden on task performance (details in Supplementary Text). The amyloid-related regional node strength measure was not significantly related to PA (permutation *P* = 0.2068).

Next, in a subgroup of participants with follow-up data available, we further tested the relationship between PA and the DMN–DAN network connectivity, based on the amyloid-related connectivity edges (Fig. 3C). The mean interval between baseline and follow-up acquisitions for this subgroup was 4.78 years (SD = 0.23, 4.42–5.20 years). Longitudinal analyses showed that baseline PA was significantly associated with changes in DMN–DAN connectivity over time (RH-DefaultC averaged connectivity with LH-DorsAttnA, LH-DorsAttnB and RH-DorsAttnB: *r* = 0.4573, *P* < 0.0490; LH-DefaultC and RH-DorsAttnB: *r* = 0.5272, *P* < 0.0204; *N* = 22; Pearson correlation controlling age, sex and the corresponding baseline connectivity).

## Discussion

In this study, we investigated how Aβ burden relates to task-modulated connectivity during distinct phases of a WM task. We found that higher Aβ was specifically associated with reduced connectivity between the DMN and DAN during the probe (WM retrieval) phase, but not during encoding or retention phases. At the regional level, Aβ burden was linked to lower node strength in posterior–medial (PM) network regions, including the PHC and Rsp, and this mediated the relationship between Aβ and WM task performance. Furthermore, we observed a significant relationship between Aβ-related network connectivity and PA, indicating a potential pathway through which lifestyle factors may support cognitive reserve in the face of early Alzheimer’s disease pathology. Together, these findings identify a phase-specific task connectivity mechanism through which Aβ disrupts WM processes and suggest that lifestyle factors, such as PA, might help promote cognitive reserve.

WM refers to cognitive processes responsible for temporal maintenance and manipulation of information, involving both attentional and executive control.^28^ WM impairment is present in the very early stages of Alzheimer’s disease and has been widely studied in normal ageing and across the Alzheimer’s disease continuum.^29,30^ In this study, we found that Aβ burden was specifically associated with altered task-modulated connectivity during the probe phase of a WM task, but not during encoding or retention. The probe phase of WM involves complex functional subprocesses, including stimulus identification and recognition, WM readout, decision-making and motor response, where the participants are required to retrieve information from the WM system and allow it to acquire control of behaviour.^51,52^ The selective association of Aβ with probe-phase connectivity may reflect amyloid-related disruptions in the neural mechanisms supporting WM retrieval. Similar task phase-specific findings have been reported for episodic memory retrieval, where Aβ was related to decreased connectivity only for the retrieval phase, not the encoding and maintenance phases.^53^ Our results suggest a potential shared Aβ vulnerability of memory retrieval during both episodic memory and WM, likely impacting through the episodic buffer component of WM.^54^ We did not observe a significant effect of memory load on network-level connectivity. This null finding should be interpreted cautiously. One possibility is that the study was underpowered to detect load-dependent connectivity effects after correction for multiple comparisons across a large number of network pairs. In addition, prior work using the same WM task has shown load-dependent changes in task activation,^55^ suggesting that memory load effects may be more strongly expressed at the activation level than at the level of network connectivity in the present sample. Future studies with larger samples will be needed for determining whether memory load introduces changes in task-related connectivity.

The PHC and Rsp are considered the PM network, which plays an important role in human memory, especially for recollection-based memories, memory for spatial and episodic context and scene perception.^56^ We found that higher amyloid was associated with reduced connectivity among the PHC, Rsp and inferior parietal lobule. This aligns with previous reports showing that Aβ deposition preferentially affects the PM networks.^57-59^ At the network level, we found that higher amyloid was linked to reduced connectivity between the DMN and both the DAN and SMN networks. This aligns with prior work showing that Aβ pathology disrupts the DMN.^8,60^ Additionally, a loss of resting-state functional connectivity between the DMN with the DAN and SMN has been related to the progression of Alzheimer’s disease.^61-63^ Together, our results extend these findings by demonstrating that Aβ-related disconnections emerge not only at rest but also during WM task execution and specifically during the retrieval phase.

At the behavioural level, our cross-validation analyses showed that amyloid-disrupted regional node strength and network-level connectivity can both significantly predict task performance. However, mediation analyses revealed that only regional node strength significantly mediated the relationship between Aβ burden and task performance. These results support previous studies showing that Aβ or neurodegenerative pathology interacts with brain connectivity to influence cognitive performance.^32,53,58,64-66^ While our sample in the current study is dominated by amyloid-negative, cognitively normal individuals, previous studies suggest that there exists a nonlinear relationship between Aβ and brain function,^32,66^ which likely varies across the stages of the Alzheimer’s disease continuum.^22,58^ For example, increased DMN–DAN connectivity was related to compensation in cognitively normal groups, whereas this hyperconnectivity also represented worse memory in an MCI group.^64^ Future work is needed to examine the relationships between Aβ burden, task connectivity and task performance at different stages of the Alzheimer’s disease continuum.

Based on prior literature, compensation refers to inter-individual variability in the ability to offset pathology-related disruption of standard processing networks through the recruitment of alternative brain structures or networks.^67^ In the present study, this concept is relevant to interpreting whether amyloid-related alterations in task-modulated connectivity reflect maladaptive disruption or compensatory network reorganization in support of WM performance. Based on the cognitive reserve and resilience framework,^13^ in the present study, ‘resilience’ refers to a general term capturing the capacity of the brain to maintain cognition and function with ageing and disease. Within the cognitive reserve framework, task-based fMRI has been used to identify functional networks that may buffer the impact of age-related brain changes on cognitive performance. For example, prior work identified a task-invariant cognitive reserve network across 12 task-based fMRI paradigms.^26^ Expression of this network was associated with IQ and moderated the relationship between cortical thickness and reasoning performance. These findings suggest that task-based fMRI, when examined within the cognitive reserve framework, can help identify neural mechanisms that support cognitive reserve and resilience in ageing. In this study, we focused only on the relationship between task-related connectivity and proxies of cognitive reserve. Further work using study designs and analyses based on the cognitive reserve and resilience framework^13^ will be necessary to determine whether these connectivity features represent a neural implementation of cognitive reserve.

Aβ deposition has been consistently linked to alterations in brain activation and connectivity. However, it remains challenging to interpret whether changes in connectivity represent vulnerability to Aβ pathology, adaptive resilience or both.^68^ However, disentangling these effects is critical for both biomarker development and intervention design.^68^ For example, some studies proposed altered connectivity as compensatory processes,^69,70^ whereas others view such alterations as detrimental network breakdown.^1,8^ To address this, we examined whether Aβ-associated connectivity was related to cognitive reserve proxies. We found that the altered network-level connectivity was associated with PA, a lifestyle factor contributing to cognitive reserve. However, this connectivity was not significantly related to two other proxies of cognitive reserve (education and IQ),^9^ suggesting that lifestyle-driven cognitive reserve mechanisms may operate through distinct neural pathways. This is consistent with previous reports, suggesting that social factors, such as education, likely act on brain networks independent of those affected by Aβ.^71^ Our results emphasize the need to incorporate such cognitive reserve variables when examining the relationships between Alzheimer’s disease pathology and brain function. In this study, we found that altered network-level connectivity was associated with PA, a lifestyle-related cognitive reserve proxy, but not with education or IQ, which are commonly used cognitive reserve proxies. This specificity warrants careful interpretation. Prior work has emphasized that cognitive reserve is an active and multifactorial process and that different cognitive reserve proxies may contribute through distinct pathways.^9^ One possible explanation is that PA reflects a more dynamic and modifiable aspect of cognitive reserve, whereas education and IQ may represent more stable, accumulated proxies of cognitive reserve.^72^ Amyloid-related connectivity may be more sensitive to lifestyle-related resilience than to mechanisms indexed by education or IQ.

PA is one potential modifiable factor for reducing the risk of cognitive decline and dementia^12,73^; however, pathways underlying its contribution of resilience against developing dementia are unknown. PA has been associated with better cognitive function,^74,75^ as well as protective effects against amyloid-related cognitive decline and neurodegeneration independent from vascular risk.^11^ However, reports on associations between PA and Aβ are often mixed.^76,77^ For example, studies showed PA benefit cognition mediated by reduction in Aβ,^78^ whereas some studies found the relationship is influenced by APOE ε4 status,^79-81^ and evidence also suggests that the associations are likely stronger in the very early phase of Alzheimer’s disease.^82^ PA has also been related to tau pathology.^83^ More recent studies have shown that PA provides resilience against the influence of tau on executive function, but exhibits no interaction with episodic memory and Aβ.^84,85^ Consistent with this literature, our results suggest that PA-related network pathways are associated with Aβ burden, which is one of the initiating pathological events in Alzheimer’s disease. However, these PA-related functional pathways did not moderate the effect of Aβ on WM performance, suggesting that they might preferentially support other non-memory cognitive domains, such as executive function. Future studies are needed to test this possibility.

Our results provided evidence that Aβ might trigger network reconfiguration, where the altered DMN–DAN connectivity represents a potential pathway of PA on brain function. However, the connectivity did not significantly moderate the influence of amyloid on task performance, which is consistent with literature showing no interaction between PA and amyloid on cognition.^84^ Previous studies have reported that PA is linked to stronger connectivity in the DMN, salience and control networks.^10,86-89^ PA has also been closely linked to couplings between the DMN and the FPN, where the DAN is also named the dorsal FPN.^90^ An intervention study showed that 12 weeks of aerobic exercise increased connectivity within posterior DMN regions, as well as between posterior DMN regions and the left postcentral gyrus of the DAN in both cognitively normal and MCI older adults.^91^ Another randomized controlled trial found that exercise training modulated connectivity within the FPN.^92^ Increased DMN coupling with the FPN has been related to cognitive flexibility^93^ and creativity in older adults,^94^ and it has been proposed as an adaptive compensatory mechanism.^95,96^ In cognitively normal individuals, greater DMN–DAN coupling has been associated with better memory performance over time, possibly reflecting compensatory mechanisms, whereas higher DMN–DAN coupling relates to worse performance in MCI.^64^ This stage-dependent effect suggests a temporally compensatory resilience mechanism but eventually relates to network failure as pathology progresses.^1^ In a preliminary longitudinal analysis, we observed that baseline PA predicted changes in DMN–DAN connectivity over time. This result suggests that DMN–DAN coupling is associated with PA in the face of early Aβ pathology in cognitively normal older adults.

Studying associations between early Alzheimer’s disease pathology and brain function, as well as their relationships to task performance and cognitive reserve proxies, has important implications for developing targeted intervention design and functional biomarkers for monitoring interventional effects. For example, given that amyloid-related connectivity changes were phase specific (prominent during the probe/retrieval phase), future work could test phase-dependent or state-dependent modulation using interventions, such as TMS to improve task performance.^97^ Prior studies have shown that the exact timing of brain stimulation relative to the phase during WM tasks is critical and exhibits distinct effects on performance.^98^ These findings highlight the potential value of brain functional measures for guiding and evaluating targeted interventions that promote resilience. Future studies should test whether interventions, such as brain stimulation, can modulate DMN–DAN connectivity in supporting WM retrieval or improving cognition in ageing.

When investigating the relationship between Alzheimer’s disease pathology and brain function, most prior studies have focused on correlation-based resting-state functional connectivity or task-evoked brain activation. However, recent work has demonstrated that task-modulated connectivity captures context-dependent brain reconfiguration, which cannot be identified using correlation-based analyses of spontaneous fluctuations or task co-activations.^23^ Only a limited number of studies have examined task-modulated connectivity in studies of Alzheimer’s disease pathology. Integrating task connectivity with Alzheimer’s disease biomarkers (amyloid, tau and cortical thickness) will be crucial for understanding the neuropathological progression of Alzheimer’s disease, as well as pathways linking pathology to cognition and cognitive reserve processes. For example, based on a task paradigm involving novel and repeated scene and object recognition, a recent study examined amyloid-related brain network disruptions using task connectivity via dynamic causal modelling.^24^ Their results showed that Aβ-related alterations in the DMN drive medial temporal lobe hyperactivity and promote early tau accumulation in the entorhinal cortex, providing evidence on how Aβ-associated network-to-network interactions relate to Aβ–tau interaction. We used a computationally simpler but conceptually related approach, i.e. PPI analysis, which enables examination of whole-brain task-modulated connectivity rather than being limited to a few predefined ROIs or networks.

Characterizing connectivity at multiple spatial scales, ranging from brain regions to networks, might provide a more comprehensive view to help understand how Aβ pathology affects brain function. Recent studies highlight the importance of considering multiple spatial scales analysis when studying brain function.^99^ Our analyses at both the nodal and network levels revealed distinct results, while regional node strength mediated amyloid effects on cognition, network-level connectivity did not, but demonstrated a significant association to PA. This might suggest amyloid-related local vulnerability of DMN regions, while network-level DMN–DAN couplings represent network adaptive response in the face of Aβ. Local disruptions in DMN hub regions may have more immediate behavioural consequences than broad network disconnections. Future studies should consider multi-scale connectivity analyses to study Alzheimer’s disease pathology relationships to brain function, such as cross-scale analyses or multi-scale network models.^100^

This study has several limitations that warrant consideration. First, tau pathology was not included due to limited sample availability, as tau-PET data were only available at follow-up and not at baseline in our study. Given that tau pathology often demonstrates a closer relationship with cognitive performance and PA, and that numerous studies showing the interactions between Aβ and tau,^101,102^ future studies should incorporate tau-PET imaging. However, a recent resting-state fMRI study suggests that DMN–DAN connectivity represents cognitive dysfunction independent of tau pathology.^103^ The present study focused on Aβ pathology because it is considered one of the earliest events in the Alzheimer’s disease cascade, making it particularly relevant for understanding early brain functional changes in cognitively normal older adults. Additionally, in this study, we did not account for APOE ε4 status, which may influence the relationship between PA and Aβ burden. Second, PA was assessed using self-reported questionnaires, which are subject to potential recall bias and measurement error.^104^ Future studies should consider objective approaches, such as accelerometry, to improve reliability.

Furthermore, although our participant inclusion did not consider amyloid PET status, the sample in the current analysis was predominantly amyloid negative. Therefore, the findings should be interpreted cautiously, particularly regarding generalizability to cohorts with more amyloid-positive individuals and to populations with MCI or Alzheimer’s disease. Prior studies of resting-state functional connectivity have suggested an inverted U-shaped relationship with amyloid burden, characterized by early hyperconnectivity followed by later hypoconnectivity during preclinical Alzheimer’s disease.^105,106^ Because our sample was composed primarily of cognitively normal individuals with sub-threshold amyloid levels, the present results likely capture only an early segment of this trajectory. The observed amyloid-related reductions in task-based connectivity may not map directly onto previously described resting-state patterns. Instead, they may reflect altered task-related network modulation during an early stage of amyloid accumulation. Future studies spanning a broader range of amyloid burden will be important for testing whether the task connectivity effects observed here also follow a nonlinear trajectory. Our findings likely reflect only early amyloid-related changes in task connectivity and may not capture patterns that emerge at higher levels of amyloid accumulation, particularly given previous work suggesting nonlinear trajectories of brain connectivity changes. Thus, our results should be interpreted cautiously and should not be assumed to generalize to cognitively normal cohorts with a higher proportion of amyloid-positive individuals or to populations with MCI or Alzheimer’s disease. However, it should be noted that the threshold used to define amyloid positivity was adopted from prior literature and was based on a global amyloid measure. In contrast, the present study focused on continuous variation in regional amyloid burden. Our findings suggest that even sub-threshold regional amyloid deposition may be associated with subtle differences in WM performance and task-related brain connectivity. These results may have implications for identifying sensitive functional markers of very early amyloid-related brain changes.

Future studies with longitudinal designs are needed to clarify the temporal sequence between Aβ accumulation and brain connectivity alterations. However, prior studies suggest that these relationships may be bidirectional, forming a ‘vicious cycle’ in which pathological accumulation alters brain networks, which in turn may facilitate further pathological spread.^107^ Finally, although our exploratory longitudinal analysis suggested that PA precedes changes in brain connectivity, interventional studies will be crucial to establish causal links between lifestyle resilience factors and brain function. The current findings may inform target selection in brain stimulation aimed at promoting resilience in ageing.

## Conclusion

In summary, this study demonstrates that higher Aβ burden is associated with reduced task-modulated connectivity supporting WM retrieval. Specifically, regional nodal connectivity, rather than system-level network coupling, mediated the relationship between Aβ burden and WM performance, suggesting the nodal vulnerability of posterior–medial and default mode hub regions to early Alzheimer’s disease pathology. Network-level couplings, especially between the DMN and DAN, were associated with PA, rendering a potential lifestyle-related cognitive reserve pathway that supports brain function in the presence of early Alzheimer’s disease pathology. Together, these findings provide insight into early-stage brain functional vulnerability to Aβ burden and identify potential network targets that may inform monitoring and guide intervention strategies aimed at promoting resilience in ageing.

## Supplementary Material

fcag278_Supplementary_Data

## Data Availability

Demographic information, PET measures, brain connectivity data and custom codes generated for this study are publicly available at https://github.com/hehengda/AmyloidLTS.git. Raw and preprocessed datasets analysed during the current study are available from the corresponding author upon reasonable request.
